# English-medium instruction in Tunisia: Perspectives of students

**DOI:** 10.3389/fpsyg.2023.1112255

**Published:** 2023-03-17

**Authors:** Marii Abdeljaoued

**Affiliations:** Laboratory of Approaches to Discourse, Faculty of Arts and Humanities of Sfax, University of Sfax, Sfax, Tunisia

**Keywords:** EMI, Tunisia, Higher education, students, attitudes, internationalization, translanguaging

## Abstract

This article gives a Tunisian perspective to the ongoing debate on the adoption of English-medium instruction (EMI) across the globe and notably in the Middle East and North Africa (MENA). It explores the attitudes of students toward EMI, especially in relation to French, the default medium of instruction at Tunisian universities. It also investigates the challenges that students encounter in courses mediated through English. Finally, it reports on the current EMI practices as they are conducted in the classroom setting. The article uses a mixed approach involving quantitative data collected *via* an online survey and qualitative data *via* classroom observation and note-taking. It was found that students typically held a positive attitude toward English and an awareness of its importance. They displayed a pragmatic stance as they associated English with research, technology, mobility, employability, and career prospects. While English is used as the language of the curriculum and documentation, students engage in translanguaging practices to ensure an effective dialog with content teachers and better acquisition of academic content. Given their multilingual repertoire and the status of French, students used French and English in parallel and, to a lesser extent, Tunisian Arabic. They tended to switch to French to ensure a more effective classroom exchange, especially when English fails them. Teachers used translanguaging in order to promote students’ engagement in the academic content.

## 1. Introduction

English as a medium of instruction (EMI) is the flagship of the unbridled globalization of economies and the internationalization of education. The implementation of EMI or English as the *lingua franca* of academia (Salomone, 2015) is a needed asset and an established practice at universities worldwide. Substituting medium of instruction (MOI) with EMI is “a relatively simple and cheap solution to both the problems of internationalization and upgraded local language proficiency” (Hamid et al., 2013, p. 11). It seems that EMI and internationalization go hand in hand, in the sense that they propel each other. Internationalization is defined as “the process of integrating an international, intercultural or global dimension into the purpose, functions or delivery of post-secondary education” (Knight, 2003, p. 2). Over the years, EMI has become more prevalent in higher education institutions (HEIs) worldwide. It is no longer a vogue but a necessary currency to join the wagon of unrestrained internationalization. It is also necessary to keep abreast with international standards, gain visibility and recognition, increase ranking in a highly competitive global market, and attract international students. Such a trend is unlikely to be reversed in the foreseeable future, and EMI is likely to gain more ground. Already, Macaro (2018) stressed that the adoption of EMI is an unstoppable trend, and nothing can be done “to halt the express train of EMI” (p. 300). This holds true even for countries where English does not enjoy a historical role. In Europe, for example, Pérez Cañado (2020) talked about three reasons or facets for this unprecedented promulgation of EMI. First, we have the strategic perspective, which has to do with the internalization of HEIs and enhancing the quality and visibility of education in the union. Second, pedagogical motives are based on the need to equip students with skills to survive the ruthlessly competitive global job markets with increased mobility programs. Finally, there are substantial motives that are, by definition, pragmatic. They prepare students and university faculty for the era when English is used as the *lingua franca* in scientific communication and instruction.

Oraif and Alrashed (2022) linked the proliferation of EMI programs to educational, economic, scientific, and technical developments and the growing need for intercultural communication. Such prevailing rhetoric on the hegemony of English as the language of science, trade, and technology made all stakeholders, policymakers, and educators worldwide take measures toward implementing and endorsing EMI. In Tunisia, however, despite the widespread adoption of social media and the 2011 change of the regime, Englishization and the promotion of English in education are still lagging as they face resistance from the guardians of the status quo (Abdeljaoued, 2022, 2021). Since EMI is a further step in the implementation of English, this would not go unnoticed by policymakers who have a vested interest in keeping the state of affairs. Badwan (2019) stated that “those who were against the shift to English found in this proposal a threat to the status quo and to the investment they have already made in French” (p. 32).

The conceptualization of EMI is only nascent and perhaps still unsound. Macaro et al. (2017) argued that “research on EMI, despite the widespread and growing interest in the phenomenon, is still at an adolescent stage” (p. 650). Pérez-Cañado pointed out that the “EMI research scene is still plagued with myriad problems and deficiencies” (p. 7). Macaro and Akincioglu’s (2018) study is perhaps the first serious attempt at what they called a state-of-the-art landscape overview. The article reviewed few literature works about EMI. They concluded that studies into EMI and its implementation fall under two broad categories. The first category of studies investigated how EMI affected language acquisition. The second category investigated the role of EMI in the acquisition of academic knowledge. They found little evidence that EMI programs improve the student’s English language proficiency. Indeed, the issue of whether EMI programs are effective in enhancing students’ language proficiency is still unsettled in the literature (Shohamy, 2012).

Before contextualizing the study, it is perhaps worthwhile to define EMI. The study of Macaro (2018) is seminal in the conceptualization of EMI as a label. He defined EMI as “the use of the English language to teach academic subjects (other than English itself) in countries or jurisdictions where the first language of the majority of the population is not English” (p. 19). It is also worth stressing that definitions are always narrow, constrained, and prone to criticism, and may even lead to more questions than answers. For example, Macaro’s (2018) definition has sparked more debate on the nature of EMI. The definition was criticized because it failed to specify the kind of English that should be used and also for failing to set the boundaries of EMI. Many questions are hazily answered in EMI as a research field. Hanging questions included the scope of EMI, its pedagogy, the required proficiency by students and teachers, and accepted classroom practices. Macaro’s (2018) definition also failed to answer questions about the influence exercised by the local cultural and educational setting on the implementation of EMI and the role of EMI in the mastery of English in general. Hence, the definition made things hazier. In the vein of feeling the way toward defining and demarcating EMI, Humphreys (2017) postulated that EMI should encompass countries where English is used as L1 since there are international students whose L1 is not English. In their extensive review of the definitions available in the literature, Pecorari and Malmström (2018) concluded that there are four characteristics of an EMI setting:

1. English is the language used for instructional purposes.
2. English is not itself the subject being taught.
3. Language development is not a primary intended outcome.
4. For most participants in the setting, English is a second language (L2) (p. 499).

In this article, I adopt Macaro’s (2018) widely cited definition of EMI mentioned earlier. This definition seems to be comprehensive as it underscores the need for students to be, at least, bilingual. Hence, it encompasses students in Tunisia. It is teaching scientific subjects through English where language learning is not the first aim but it can take place as a by-product of the practice.

We now move to contextualize the study. Similar to the cases reported in the literature such as Japan (Curle, 2018; Aizawa and Rose, 2020), Oman (Ali, 2020), UAE (Solloway, 2016), Iranian expatriates in Japan (Rakhshandehroo, 2017), Taiwan (Chang, 2010), France (Reynolds, 2022), Afghanistan (Orfan and Seraj, 2002), and many others, Tunisia is not an exception to this trend of the internationalization of HEIs and the promotion of EMI. Tunisian HEIs are attracting international students mainly from neighboring Libya and sub-Saharan Africa. The trend of using EMI has been making progress in leaps and bounds, especially at business schools. Tunisia’s private and public HEIs are trying to attract international students by developing new programs where students pay fees. For example, this year, the faculty of dentistry of Monastir opted for EMI. Manouba School of Engineering specializes in geomatics and ecological engineering and teaches exclusively in English. The programs are believed to be attractive to international students since the tuition rates are relatively cheaper than in other neighboring countries such as Egypt or Morocco.

Over the past years, there has been a burgeoning interest in EMI propelled by marked forces, yet the perception of the trend and the challenges faced by students remained largely uncharted. In addition, given the inchoate development in the research area, research from another sociocultural and linguistic background is always welcome in order to have a better understanding of the development of EMI worldwide. Since EMI cannot be studied in isolation from its socio-cultural context, knowing the practices in every country is worthwhile. As Shohamy (2012) stated, “there are significant lessons that can be learned from each of the settings that may have an impact the others” (p. 197). Hence, another study from another context does not seem to be a replicate. Unlike most of the literature where the choice, or perhaps the conflict, is between the vernacular language and English, in Tunisia, two foreign languages are in rivalry: English and French.

It is perhaps worthwhile to note that there is little research devoted to EMI in Tunisia. Badwan’s (2019) study is, to my knowledge, the only research about the potential of EMI in Tunisia and the readiness to shift to English. Although Badwan's (2019) study is worthwhile, it is a ground-exploring study and it did not explore the actual status of EMI. It did not use lived experience in the use of EMI in the selection criteria of candidates for the questionnaire or the interview. Respondents are not affiliated with Tunisian HEIs where courses are administered in English. Hence, little is known about the student’s perceptions of non-language subjects taught in English in Tunisia. In addition, students’ experiences of making the radical transition from French-medium instruction to EMI are not explored. This study is perhaps a timely contribution as it adds to the body of knowledge about EMI by offering a Tunisian perspective to the debate about EMI implementation in the MENA region. It is also pertinent to recall that research into EMI is scarce not only in Tunisia but also in Africa and the MENA region. In the words of Alhassan et al. (2021), “there is little attention given to EMI in the Middle East and the North and sub-Saharan African contexts” (p. 1). Hence, this study and the whole issue seem to be a needed contribution to the literature.

## 2. Literature review

### 2.1. English and EMI in Tunisia

Tunisia belongs to the MENA region. It is an ex-French colony that is still trying, against all odds, to distance itself from French rule and the French language and embrace English as a tool for development and growth. Although English does not enjoy the history of French in the linguistic landscape of the country, it is gaining ground in the education system and the debates about the language policy orientations of the country. With the rise of corporate culture, globalization, and the mushrooming mass media, English has been ubiquitous in all aspects of life. It is incredibly fashionable with the Z-generation (Abdeljaoued, 2023). Tunisia was ranked first in the Maghreb according to the English proficiency index (version 2021)^1^ issued by the international educational institution “Education First.” The language landscape in Tunisia has often been characterized by the rivalry among Arabic, French, and English. The language policy has also lacked vision and sustainability since political agendas have always had the upper hand over the educational and pedagogical needs of the students. One example of such a capricious and absurd policy is that scientific subjects are provided in Arabic at the junior school and then it switches to French at the high school. English is taught as a second foreign language in Tunisia, after French. The provision of English starts from the fifth grade of basic education in public schools and much earlier in private schools. Students study English in secondary education until they are awarded the Baccalaureate diploma (a school-leaving diploma equivalent to GCSE). While teaching French as a compulsory subject stops with baccalaureate, English continues to be present in all universities in the form of ESP courses. Abdeljaoued (2021) stated that these courses are mostly distorted hodgepodge forms of ESP and a replicate of content courses.

In Tunisia, although there are no explicit policy directions on the language of instruction in tertiary education, French has always been the default language of instruction except for some humanities and, naturally, students majoring in English and Arabic. Using EMI in tertiary education and developing a legal framework for its use has always been an aspiration for generations in Tunisia. However, due to what Sabaté-Dalmau (2020) called “whispers of resistance to EMI policies” (p.70), little has been done in that respect despite the humble isolated attempts. Locally, there have always been many dynamics at play in the decisions about HE policies, and education policy in general, in the country. In addition, there seems to be a psychological barrier (Badwan, 2019). Since content teachers are only familiar with French as a medium of instruction and research, it is difficult for them to switch to English; although, they might be convinced that EMI is more appropriate now.

English-medium instruction is relatively new in Tunisia and it is en vogue mostly in business studies. Tunis Business School is the first HEI to offer business courses in English. It was officially established in 2010. It is the first and only public business institution in Tunisia to use English as the language of the instruction following the U.S. higher education academic system. In 2022, Manouba School of Engineering was established as the pioneering public school to offer engineering courses taught in English. Similar to Tunis Business School, Manouba school of Engineering targets students who enjoy strong potential and have a good command of English. The school is also open to international students from English-speaking countries as well as Sub-Saharan Africa. The private sector followed suit and offered more structurally organized academic courses in English.

It is worth noting that the public sector failed to provide education at acceptable standards mainly due to problems with infrastructure, untargeted recruits, crowded classrooms, and conflicts with education syndicates (Abdeljaoued, 2022). Tunisians who can afford the fees are turning to the private sector where their children can have a comfortable education. This high demand has sparked investors’ interest in education as a new yet highly profitable sector (Abdeljaoued, 2022). As for the year 2022^2^, private higher education comprises 76 HEIs (27.4% of all HEIs in the country). The number of students in the private sector is 42,241 (14.1% of all students in the country). The sector also attracted 5,075 international students mainly from Sub-Saharan Africa (61% of all international students in the country). These statistics show that not only is the private sector gaining ground (given the conditions in the public sector and the relatively low fees compared to other countries) but also the favorite destination for international students.

### 2.2. Students’ perception of English and EMI

The most covered theme of research on EMI is the focus on the perception of both students and teachers (Lei and Hu, 2022). It is followed by research on the impact and practices of EMI. The foci of research on students’ perception included the attitudes toward EMI, the motivations, and satisfaction with the provision of content using EMI. Another worthwhile focus was the resistance to EMI. A newcomer is a burgeoning interest in international students’ perception of EMI. It included how international students perceive EMI and the content teachers use in English.

Students worldwide expressed overall satisfaction with the use of EMI. For example, Kuteeva et al. (2015) reported the students’ statistically had a positive attitude with an awareness that is the *lingua franca* of the business market. Another key finding in this study is that while Swedish students were so confident with their use of English that they could use it naturally, international students in Swedish HEIs reported some anxiety in the use of English. Surprisingly, to the researchers, Swedish students stated that overall, their English was better than that of their content teachers. Another key finding, perhaps not so for the researchers, was that Swedish students find it natural to study in English and they tended to disagree that EMI is challenging. Similarly, Spanish students in Lasagabaster’s (2022) study were positively inclined toward EMI. They also reported the new horizons and career prospects that EMI opens for them. Nevertheless, unlike their Swedish peers, Spanish students were not as confident in using EMI. They reported that EMI, although important, tends to make learning more complex. They also linked the proficiency and easiness of language use and less command with more worry. However, they believed that EMI has a very positive impact on the development of the four language skills, even in those contexts where students’ language proficiency is low. Spanish students at the University of the Basque Country (Doiz and and Lasagabaster, 2018) reflected upon their experience with EMI. They underscored the importance of EMI, especially for management where the terminology and literature are primarily in English. The student’s learning experience of EMI is good. They were comfortable and happy with their academic courses in English and they even asked for more subjects to be mediated through English.

It is a truism that all students share the symbolic and economic capital of English. Yet, English language proficiency varies from one country to another. The great confidence in the use of English that Swedish and Spanish students showed is not shared by their counterparts from other socio-cultural contexts. For example, Korean engineering students in Kim et al.’s (2017) study felt deficient, even vulnerable, about their English ability, with a marked preference for Korean-medium instruction over EMI. They also did not feel the correspondence between EMI and their English ability. Like Swedish students, Korean did not report harm in translanguaging in the classroom. This is potentially a good solution when EMI fails.

Nevertheless, unlike Swedish students, Korean students evaluated their content instructors’ ability positively and sometimes highly favorably in the use of English. Although Swedish people and Scandinavians in general, have always been highly profiled in the use of English, the students were somehow critical of their teachers’ mastery of English. Denman and Al-Mahrooqi (2019) studied the perception of students and faculty at Omani universities and focused on the experience of translanguaging and the potential effect of EMI on Arabic identity. Participants identified many advantages of EMI, including increasing employment opportunities and facilitating communication in education and workforce settings. However, significant challenges were also reported. Problems such as the limited acquisition of course content when delivered in English were recurrent. Teacher participants did not believe EMI had any adverse effects on learner identity and Arabic language use. However, students were neutral and maintained that their assimilation of course content would be enhanced if mediated through Arabic. Chinese students in Kong and Wei (2019) generally favor using EMI. However, they lacked consensus on the importance of EMI, as 40% were either undecided or disagreed with the statement. In addition, 70% of the respondents thought that EMI helps improve their English. This result should be taken with caution since the perception of improvement cannot be confirmed without tests before and after the provision of EMI. Afghan students in Orfan and Seraj (2022) had a reasonably positive attitude toward EMI. Over 70% of the participants linked English to high status. They also believed that Afghani society generally thinks highly of people who study in EMI programs. Moreover, more than 80% of the participants’ English (and EMI) is more prestigious. Another key finding was that 72% of the participants stated that taking courses in the medium of English was essential even if they got low grades. Already, Tunisian students in Badwan (2019) agreed most strongly that they are ready to bring their English to the requested level needed for EMI courses if their teachers swap to English. A majority of 85 students agreed that EMI would make their university diplomas more recognized worldwide.

Translanguaging as a pedagogic practice is a psycholinguistic and sociolinguistic theory that is understood as a function of bilingual identity. It investigates the meaning-making practices of multilingual students and teachers (Kleyn and García, 2019). Hence, it is part of people’s translanguaging identity and it can be broadly defined as the use of the full range of languages and semiotic resources in order to create meaning and negotiate classroom content (Li, 2018). The translanguaging theory postulates that multilingual students have an integrated repertoire of linguistic and paralinguistic resources from which they draw during classroom encounters. García and Wei (2014) mentioned two main types of classroom translanguaging: student-directed and teacher-directed. Both approaches have added pedagogical value in bilingual or trilingual instructional settings. They are used for scaffolding students, promoting dialog with content teachers, and negotiating academic content.

### 2.3. Students’ challenges toward using EMI

English-medium instruction is a boon and a bane since it can bring benefits, and it can also inflict challenges and extra load for non-Anglophone students. EMI has always been an educationally contentious issue. Non-Anglophone students all over the world have reported a whole slew of challenges that they face while using English to study academic subjects. In their review of the related literature about student and content-teacher challenges, Lei and Hu (2022) identified an array of challenges in EMI. They ranged from language difficulties, workload, extra time needed to prepare lectures in English, and additional time needed for studying. Content teachers’ lack of competence in using English is a challenge that students have to deal with. Already, as Doiz and Lasagabaster (2018) aptly stated, EMI takes place in university contexts in which many of the professionals teaching EMI are nonnative speakers teaching primarily nonnative students, “a situation that more often than not contributes to a sense of insecurity among all the stakeholders” (p. 658).

One of the most prevalent challenges reported in the literature was the students’ low proficiency. Related literature works such as Tri and Moskovsky (2021), Brown (2017), and Kim et al. (2017) agree that student’s low proficiency is the root cause of the failure of courses mediated through English. Non-Anglophone users of EMI could not grasp content knowledge in English and failed their writing tasks due to the language barrier. Turkish students in Kamasak et al.’s (2021) study found writing and speaking incredibly daunting. This is quite understandable since these skills are productive and call lexical, grammatical, and communicative competencies into play. It is a truism that this can only be a source of frustration, even demotivation, anxiety, and fear of losing face. Other researchers evoked the disappointment theory when talking about students’ challenges when using EMI. Students in Reynolds (2022) reported that their disappointment is most marked when they compare themselves to more proficient peers. This was one of the most serious criticisms leveled against EMI. Shohamy (2006, 2012) stated that the huge gap in language proficiency undermines the chances of equal opportunities as the premises of the learning experience. Hence, it discriminates against students with low proficiency. The discrimination and biases are even more marked due to the assessment in a second language. She adds that it is not only the right of equal opportunity which is at stake but also the acquisition of academic knowledge. Similarly, Hu et al. (2014) expressed their surprise when finding out that the adoption of EMI “tended to perpetrate and accentuate inequalities” (p. 37). Within this context of self-doubt and disappointment, a thorny problem was the lack of writing abilities and communication skills. The related literature agrees that communication, including oral presentations and taking part in activities and debates in class, was frustrating for students. This is because their English falls short of expressing their ideas. This can only deter students from speaking and hamper a fruitful teacher–student interaction. Doiz et al. (2011) described the situation by stating that students do not ‘*dare*’ due to feelings of restraint, anxiety, and intimidation. Solloway (2016) talked about Emirati students’ fear of using English in front of their peers and the artificial nature of using an L2 with speakers of the same L1 (Arabic in this case).

English-medium instruction is equally challenging for international students. Volchenkova and Al-Darraji (2022) classified the challenges faced by international students into four categories. The first is linguistic challenges which include all manifestations of the lack of language proficiency; the second is academic challenges which include EMI academic traditions, pedagogical methods, and assessment practices which are at odds with the practices they are familiar with in their home countries; the third is cultural challenges which include discrimination, bias, and cultural adjustment challenges; and the fourth is social challenges which include new (hence strange) communication modes, limited social life, and adapting to local food and transportation. In Hellekjær's (2010) comparative study, Norwegian students did not report serious difficulties with EMI instruction. German students, however, reported some difficulties in assimilating EMI course concepts and objectives.

Although expected, incidental language learning seems less-than-desired outcomes. Acquiring English was just an offshoot or a byproduct of assimilating academic knowledge. This offshoot nature drove Pecorari and Malmström's (2018) definition of EMI. As they stated, “EMI is a setting in which English skills are not specified as a curricular outcome, are rarely planned for, and are not systematically taught, but which are nonetheless expected to be acquired” (p. 502). Lasagabaster (2022) traced this two-in-one nature of EMI to the fact that in EMI, unlike CLIL, there is an explicit dual focus on language and content. Yet, there is little collaboration between content and language since the focus is only on the content, and language acquisition is not assessed. However, the literature also gave evidence to the contrary. The underlying assumption is that EMI inevitably improves English language proficiency (Bolton et al., 2017). Extensive exposure to the target language leads to “opportunities for meaningful use of it to negotiate the curricular content, thus leading to better acquisition” (Galloway et al., 2017, p. 6).

Using English to teach scientific subjects comes with some shortcomings and challenges. Teachers’ below-par level of English was especially problematic for students. These challenges stem from the context where EMI is implemented and not from the implementation of EMI itself. Both students’ and lecturers’ low English skills can seriously threaten the flow of courses mediated through English. There are two fundamental problems with lecturers’ low English proficiency. First, they can cause teacher anxiety and self-doubt. Second, as reported by Salomone (2015), it can hamper student–teacher interaction and reduce classroom activities’ naturalness and smoothness. In this respect, it is pertinent to recall the ‘demotivation and disappointment’ theory. This is perhaps a fundamental question in the research and conceptualization of EMI.

According to Tang and Hu (2022), student demotivation with English as a medium of instruction has attracted increased attention from scholars, particularly in countries where English is taught as a second or foreign language. While there is a consensus that proficiency in English brings several benefits, it is found that students are demotivated to learn in English. Badwan (2019) talked about the potential (expected) challenges that Tunisian students might face if universities swap to English. Surprisingly, students ranked content teachers’ unreadiness to use EMI as the top challenge, followed by the student’s failure to understand the content subject due to the language barrier. Another worthwhile finding was that the students were aware that it would be possible to discuss subjects in class and write their assignments in English. Badwan’s study hypothesized about using EMI in Tunisia; it is not based on EMI experiences, discussions of lived experiences, and felt challenges in the EMI classroom.

### 2.4. Research questions

The study is a case study of student attitudes toward EMI and classroom practices where English is used to study academic subjects. More specifically, it seeks to report and analyze students’ attitudes toward EMI at Tunisian HEIs and the practical aspects of teacher–learner interaction in an EMI context. The study sets out to address the following four research questions:

RQ 1: How do Tunisian students perceive the use of EMI?

RQ2: How do students in Tunisia perceive English in relation to French and Arabic?

RQ 3: What challenges do Tunisian students experience in EMI provision?

RQ4: What translanguaging practices are there in the provision of academic courses in English?

## 3. Methodology

### 3.1. Student survey

The study was conducted in Tunisia HEIs, both public and private. It sets out to discover the attitudes and experiences of EMI within HEIs in Tunisia. Unlike Badwan (2019), this study targets students with lived experience in EMI programs. Badwan’s British Council commissioned a study reported on the potential provision of EMI and investigated the readiness of university teachers, students, and educational stakeholders to move to EMI programs. The respondents had no experience in EMI provision. This research, however, will report on the lived experiences, perceptions, and challenges based on contact with EMI rather than hypothesizing about the potential use and expected challenges of EMI use. This study opted for a quantitative-qualitative approach to research using snowball sampling.

The questionnaire was designed trilingually showing in English, French, and Arabic in order to ensure the understanding of the questionnaire items.

The data for this study were collected from computing, engineering, business, and management courses provisioned in English at the Tunisian HEIs (both private and public). The dataset consists of a student survey (*n* = 434) focusing on student perception of EMI and challenges encountered in learning content subjects *via* English. Since this study is perhaps the first to delve into the issue, a survey is well-suited as a data collection method because it allows access to a significant mass of the target population; hence, it helps glean a massive amount of information. According to the related literature, the questionnaire is ideal for exploring participants’ attitudes, opinions, beliefs, and practices. According to Dörnyei (2003), “questionnaires can yield three types of data about the respondent: factual, behavioral, and attitudinal” (p. 8). Factual questions range from demographic characteristics, subject descriptors, socioeconomic status, and level of L2 proficiency. Behavioral questions describe people’s actions, lifestyles, habits, and personal histories. Attitudinal questions find out what people think. Under this category, we find attitudes, opinions, beliefs, interests, and values (Dörnyei and Taguchi, 2009, p. 6).

The student questionnaire was based on the related literature. It was also partly informed by my teaching experience with a portion of the target population and informal discussions with content teachers. It consisted of four main sections. The first section elicited demographic and background information. Section two reports on the respondents’ level of English. On a 5-point Likert-type response scale, students are asked to self-rate their level of proficiency in English. Response options ranged from strongly agree to strongly disagree, with a neutral mid-point. Section three is about the perception of English-Medium Instruction. Finally, section four reports on the current status of EMI and practice and challenges as perceived by students.

The questionnaire was designed online using the Google survey tool to collect data. It was written in English, French, and Arabic to ensure maximum understanding of the survey items. The link to the questionnaire was shared with lecturers in many HEIs using personal contacts and the database of contacts available on each institution’s site. Content lecturers as well as administrative staff of the HEIs delivering courses exclusively, or partially, in English were requested to share the survey link with the students. In addition, students who filled out the survey were asked to recruit their peers. In compliance with the ethical code of conducting surveys, students were asked to read the purpose of the study and the consent statement. The students were assured of their rights to anonymity and confidentiality of personal information. However, Google forms are already designed to blind information such as names and email addresses of the researcher.

### 3.2. Classroom observation instrument

In order to offer a thoroughgoing picture of EMI practices in Tunisia, this study opted for classroom observation to triangulate the survey data and get better insights into EMI class interaction other than self-report. The classroom observation instrument focused on classroom talk and the negotiation of the academic content of the lesson. The two content teachers who use EMI in my institute granted their consent to be observed in the classroom. Each teacher had a lesson per week so class observations were conducted over 2 weeks of semester 1 of the academic year 2022–2023. Four classroom observations were made; each session lasted around 75 min. This produced around 5 h of naturally occurring classroom talk. In addition to notice-taking, I videotaped the lesson because it is perhaps hard for any researcher to observe everything. The classroom context also enabled me to observe how English, Arabic, and French are used in the classroom, especially with regard to code-switching between French and English (Bach Baoueb and Toumi, 2012; Lewis et al., 2012).

### 3.3. Data analysis

For the online survey, I analyzed students’ answers using descriptive statistics. Answers to the survey questions in a Linkert scale were uploaded to Google spreadsheets to analyze them and get descriptive statistics. The quantitative data were reported in tables and figures to compare the results more easily. Descriptive statistics measured participants’ attitudes toward EMI, its importance, as well as classroom practices. The components of descriptive statistics were used to present the quantitative data, including frequency, percentages, and central tendencies (mean and standard deviation). Finally, a statistician validated the results.

The classroom interactional data are analyzed using Multimodal Conversation Analysis (MCA) to document naturally occurring classroom interactions. MCA has the analytical power of exhibiting the detailed and complex process of how translanguaging practices are realized in the classroom. MCA provides a participant’s perspective on the data by revealing how classroom talk is realized in turns using multi-modal mediums. It also analyzes how participants in the communicative event achieve mutual understanding in interaction (Mondada, 2018; Querol-Julián, 2022).

### 3.4. Population

Any research needs to start with the respondents’ profiles since demography can account for the orientation of the respondents’ answers. Table 1 reports the demographic information about the students who responded to the survey. As shown in Table 1, most of the respondents are female students. This is, in fact, not surprising since there has long been a disparity between male and female students at Tunisian universities. Although official statistics have always denied this fact; yet, any teacher or visitor to any Tunisian HEI will see that there is a disparity. Another finding is that 82% of the respondents belonged to management and business schools followed by 8% from the computer sciences.

**Table 1:** Demographics (434 respondents).

Gender		Age		Institute		L1	
Male	126 (29%)	-22	256 (59%)	Public	334 (77%)	Arabic	420 (96.8%)
Female	308 (71%)	≥ 22	178 (41%)	Private	100 (23%)	Other	14 (3.2%)
Nationality		Major					
Tunisian	426 (98.2%)	Management	357 (82.2%)		Computer Sciences	34 (7.9%)	
Other	8 (1.8%)	Engineering	26 (6%)		Economics	17 (3.9%)	

## 4. Results and discussion

### 4.1. Reports of English proficiency

It is a truism that competence in any language can affect people’s perception of the language since proficiency tends to make users of the language perceive it favorably. It seems that there is a causal relationship between the students’ fairly high proficiency in English and their positive attitude toward EMI. To my surprise, most of the respondents reported high proficiency in the use of English since the mean value of general competence was 3.76/5 and a low standard deviation of 0.98. This means that the proficiency level is clustered around the mean value of 3.76 (good to very good). This is confirmed by the fact that 84.9% of the students rated their English as good, very good, and excellent. Only a negligible 11 respondents (2.5%) rated their general English Proficiency as poor. Although this finding is surprising, it is perhaps not difficult to explain it. Since, mainly in the public sector, a good mastery of English is a pre-requisite requirement for admission at the Tunisian public schools offering EMI courses. In addition, candidates are selected based on an admission test where the student’s level of English is tested. This explains, to a great extent, the respondents’ confidence in their proficiency in English. Nevertheless, confidence level varies quantitatively when we go from one skill to another.

The sample was most confident in their reading skills, with 94.2% of the respondents rating their reading abilities as ‘good’ or above, including 41.9% who thought their reading in English was excellent. However, only 18% of respondents rated their writing capabilities as ‘excellent’, although 87.4% of respondents still thought their writing skills were good or above. Rating their pronunciation abilities, 20.5% of students said their pronunciation in English is ‘excellent’ and 86.4% rated their pronunciation as ‘good’ and above. English grammar is the least rated under the ‘excellent’ category, with 14.1%, but 86.4% still believed their mastery of English grammar is, at least, good. The respondents showed a significant degree of confidence in their English proficiency. Just by looking at the mean value of responses, we notice the high degree of self-rated English proficiency (Table 2).

**Table 2:** Self-rated English language proficiency.

Number of respondents giving each answer							
Item	Excellent (5)	Very good (4)	Good (3)	Fair (2)	Poor (1)	Mean	SD
7. How do you rate your English overall proficiency?	104	178	108	33	11	3.76	0.98
8. How do you rate your English reading ability?	182	147	80	19	6	4.1	0.94
9. How do you rate your English writing ability?	78	157	144	45	10	3.57	1.05
10. How do you rate your English speaking ability?	97	156	120	43	18	3.62	1.05
11. How do you rate your English pronunciation?	89	134	122	42	17	3.58	1.07
12. How do you rate your English grammar?	79	145	141	55	14	3.5	1.04
13. How do you rate your English vocabulary?	61	166	148	46	13	3.5	1.0

### 4.2. Attitudes toward English and EMI

This section answers the first research question pertaining to the perception of English and EMI. The research question pertains to the respondents’ perception of EMI, especially in relation to French, the default medium of instruction, and to a lesser extent, Arabic. Table 3 reports the number of students in each option to perception questions along with corresponding mean responses and standard deviations. There is a high level of agreement about the status of English and its utility in the future. Item (15), ‘I will need English in my future career’, received the highest mean value of 4.42 and a small standard deviation of 0.96, which shows that most responses are clustered around the mean. This shows students’ awareness of the status of English and its linguistic capital as the *lingua Franca* of science. Even when they perceive it unfavorably, they are aware of its utility. Item (14), ‘English is the language of science, technology, and communication’, received the second highest mean value at 4.28 with 86% of respondents agreeing to the statement and only 5.5% disagreed. A related item, ‘EMI programs offer students more employability opportunities’ received the highest mean (4.53) and very low standard deviation (0.74). The respondents joined other students in the germane literature in highlighting the importance of EMI in fostering employability. Items (17–19) are pertinent to the respondent’s perception of the language policy in Tunisia and languages in contact in the country. For example, Item 16 seeks to see whether the respondents perceive English as a threat to Tunisian culture and traditions. The mean value of responses falls at disagreeing, with 69.8% of students disagreeing and 17.7% without opinion on the issue, and 12.4% of respondents agreeing. The respondents joined peers from UAE (Solloway, 2016) who did not see the need for English as a threat to national identity. Yet, respondents in Reynolds (2022) had different opinions. This is quite understandable given the fierce rivalry between French and English. Given also the fact that the French language for France is not just a language but the spearhead of its foreign policy around which the Francophony and its survival are built.

**Table 3:** Language and EMI perception.

Number of respondents giving each answer							
Item	1*	2*	3*	4*	5*	Mean	SD
14. English is the language of science, technology and communication	16 (3.7%)	12 (2.8%)	34 (7.8%)	142 (32.7%)	230 (53%)	4.28	0.98
15. I will need English in my future career.	20 (4.6%)	5 (1.2%)	15 (3.5%)	68 (15.7%)	326 (75.1%)	4.42	0.96
16. EMI programs offer students more employability opportunities	4 (0.9%)	8 (1.8%)	19 (4.4%)	124 (28.6%)	279 (64.3%)	4.53	0.74
17. English language is a threat to Tunisian culture and traditions	161 (37.1%)	142 (32.7%)	77 (17.7%)	46 (10.6%)	8 (1.8%)	2.07	1.06
18. English is a threat to French language and France’s interests in Tunisia	72 (16.6%)	66 (15.2%)	97 (22.4%)	126 (29%)	72 (16.6%)	3.2	1.29
19. I perceive French rather negatively because of the colonial history of France in Tunisia	69 (15.9%)	107 (24.7%)	107 (24.7%)	81 (18.7%)	70 (16.1%)	2.9	1.3
20. I think offering courses in English has attracted foreign students to study in Tunisia.	10 (2.3%)	8 (1.9%)	50 (11.6%)	192 (44.4%)	172 (39.8%)	4.16	0.99
21. EMI is an authentic way to learn English	9 (2.1%)	20 (4.6%)	51 (11.8%)	162 (37.3%)	192 (44.2%)	3.76	1.3
22. EMI programs help improve students’ English proficiency	9 (2.1%)	6 (1.4%)	21 (4.8%)	170 (39.2%)	228 (52.5%)	4.38	0.81
23.Tunisian universities should swap from French to English as medium of instruction	28 (6.5%)	21 (4.8%)	46 (10.6%)	134 (30.9%)	205 (47.2%)	4.14	1.18
24. I think it does not make a difference what language the teachers use	99 (22.8%)	186 (42.9%)	78 (18%)	54 (12.4%)	17 (3.9%)	2.31	1.13
25. I prefer to be taught all subjects in French	185 (42.6%)	158 (36.4%)	64 (14.7%)	21 (4.8%)	6 (1.4%)	1.85	0.96
26. I prefer to be taught all subjects in Arabic	194 (44.7%)	116 (26.7%)	70 (16.1%)	36 (8.3%)	18 (4.1%)	2.00	1.15
27. Content teachers instill positive attitudes toward EMI into their students	20 (4.6%)	56 (13%)	127 (29.4%)	148 (34.3%)	81 (18.8%)	3.48	1.08
28. I think teachers interact less with students in courses taught through English compared to courses in French	47 (10.8%)	100 (23%)	75 (17.3%)	138 (31.8%)	74 (17.1%)	3.21	1.27

*(1) Strongly Disagree, (2) Disagree, (3) Neutral, (4) Agree, and (5) Strongly Agree.

The following section reports students’ answers to the second question which positions English on the linguistic map against the other languages in the country: French and Arabic. This is perhaps important given the emerging triglossia in Tunisian tertiary education. Item (18), ‘English is a threat to the French language, and France’s interests in Tunisia’, seems to be a polarizing question with responses splashing out along the scale. Surprisingly, 16.6% of respondents are rated on each of the two extremes of the scale (extremely disagree and extremely agree). The mean value (3.2) falls between neutral and disagree. This means that the respondents do not have opinions on the issue. Thus, we can find credence in the fact that the Z-generation (Abdeljaoued,2023) did not witness the heated debate on language policy in Tunisia. There was a time when English was perceived negatively under the spell of French teacher-expatriates who not only transposed the French model of teaching but also transferred the negative feeling toward English to the first generations after independence. A similar case was reported by Reynolds (2022) where students’ attitudes to English and French are in keeping with the diglossic situation in France. English is gaining ground despite the historic rivalry between the two languages.

This generation of students are Internet residents; they grew up with social media and video games in English. Whether in Tunisia, France, or Algeria, they tended to bypass the sterile polarization in which their fathers were engaged. Indeed, this ideology-free perception makes sense and is confirmed by Item 18 (I perceive the French language rather negatively because of the colonial history of France in Tunisia). The results of this item are quite surprising. While almost a quarter of the respondents had no opinion, 40.6% disagreed with the statement. This set of questions is not directly linked to the perception of EMI, but it is interesting to know the respondents’ mindset. Item (20) is a classic question in the EMI germane literature (e.g., Rakhshandehroo, 2017; Curle, 2018; Alhassan et al., 2021). The students mainly agreed with this statement, with 84.2% agreeing (mean value 4.16) and only 4.2% disagreeing. This reflects a high awareness of the importance of English and its supremacy not only as the *lingua franca* of academia but also as the language of the global market. Indeed, the respondents joined their Swedish peers in Kuteeva et al. (2015) in perceiving English favorably. The mean of student responses to “EMI is an authentic way to learn English” is a relatively high mean (3.76) and a relatively low standard deviation of 1.37. This is confirmed by Item 22 (EMI programs help improve students’ English proficiency). The mean response to this item was high at 4.28, with a low standard deviation of 0.81. Although this is contentious in the literature, there is no evidence that students can boost their English proficiency through immersion in English-only classes. Yet, students felt EMI courses helped in that respect. Similarly, students responded with a high mean of 4.14 (SD = 1.18) to Item (23), ‘Tunisian universities should swap from French to English as a medium of instruction’. This again points to two facts. First, it points out that students think pragmatically since they do not seem emotionally attached to French. Second, it points to an awareness of the importance of English when used as an academic *lingua franca*. Such awareness is confirmed in Item (24) ‘I think it does not make a difference what language of instruction teachers use’. Approximately 67.7% of the respondents disagreed with the statement (mean value = 2.31, SD = 1.13). Furthermore, when the students were asked if they preferred to be taught in French and Arabic (Items 25 and 26), they responded with a low mean of 1.85 and 2.0, respectively. Compared to the literature from similar cultural backgrounds, we find that Emirati respondents in Findlow (2006) have different opinions from Tunisian respondents. In her large-scale study, Findlow (2006) found that 22% of students preferred to be taught in Arabic, 50% in English, and 28% said they want to be taught in both languages. This divergent perception toward Arabic between Emirati and Tunisian students is quite interesting. Similar to Turkey, the Westernization policy endorsed by post-independence Tunisia instilled in Tunisians a belief that Arabic is not the language of science. Although, this turned out to be a fallacy with success stories where Arabic is used as a medium of instruction. Arabic is used successfully to teach sciences in the Arabian Gulf, especially in Iraq and Syria.

Although the students showed a clear preference for English, the linguistic transition should be initially slow. Badwan (2019) asserted that an abrupt shift to English would be a linguistic leap for the students to tackle. She called for a smooth transition by offering partial EMI programs while offering a transition period of intensive English classes at the beginning of the academic programs. This seems a reasonable course of action since Tunisian students already have this linguistic leap where the teaching of science subjects (mathematics, physics, IT, etc.) abruptly shifts from Arabic at the Junior high school to French at the senior school without any preparations. The results have been catastrophic for both teachers and students.

The last two questions in this section (Items 27 and 28) yielded quite surprising results. Although the students agreed that their content teachers instilled in them a passion for English (Mean = 3.48, SD = 1.08), they similarly agreed that teachers interact less when using English compared to French (mean = 3.48, SD = 1.8). This is consistent, although it may not sound so. Most content teachers, including those who have been trained abroad, have their studies in France or Francophone Quebec. In addition, content teachers who use EMI are not selected based on their competence in English, nor are they certified to teach in English. There are also no particular pedagogical guidelines for using EMI. Similar to Vietnamese universities (Sahan et al., 2022), Tunisian HEIs encourage teachers to swap to English, without qualifying them or requiring them to be certified to teach English. Even if the ministry of higher education takes the initiative to provide in-service professional EMI training for content lecturers, there would be teacher resistance given the top-down nature of the decision, the lack of incentives, and the extra burden to deal with. Hence, content teachers tend to transpose their academic knowledge in French into English. Without such training, content teachers learn to use EMI by doing rather than focusing on pedagogical training. Even if they are offered training in EMI pedagogy, they are not ready to accept it because they are not willing to be treated as students again as respondents in Badwan (2019) said.

### 4.3. Students’ EMI challenges

This section addresses the third research question which seeks to find out about challenges in EMI programs from the perspectives of students. The students are asked to quantify their challenges in using EMI on a Likert scale. The results of the challenge are reported in Table 4. Surprisingly, EMI does not seem to be a source of extra workload for students since 66.8% disagreed on Item (29) whereas 16.4% agreed. This finding is against a large body of literature where similar questions were asked (e.g., Hu et al., 2014; He and Chiang, 2016; Kamasak et al., 2021). Let us recall that students generally reported high proficiency in English, and thanks to that proficiency, they are admitted to EMI programs. This result makes sense with this background knowledge. Another surprising finding is reported in the results of Item 29 (Students have difficulty understanding the lecture due to their teacher’s lack of English proficiency). A majority of 64.2% of students agree with this statement at a high mean of 3.75 and 1.02 SD.

**Table 4:** Student EMI reported challenges.

Number of respondents giving each answer							
Item	1*	2*	3*	4*	5*	Mean	SD
29. EMI programs create more workload for students	128 (29.5%)	162 (37.3%)	73 (16.8%)	58 (13.4%)	13 (3%)	2.23	1.13
30. Students have difficulty understanding the lecture due to their teacher’s lack of English proficiency	12 (2.8%)	47 (10.8%)	81 (18.7%)	190 (43.8%)	104 (24%)	3.75	1.02
31. Content lecturers’ English proficiency is generally better than that of students.	121 (27.9%)	118 (27.2%)	79 (18.2%)	70 (16.1%)	46 (10.6%)	2.68	1.41
32. Students find it difficult to understand textbooks in English	37 (8.5%)	159 (36.6%)	120 (27.6%)	102 (23.5%)	16 (3.7%)	2.77	1.01
33. I find it difficult to structure and put my thoughts in English	145 (33.6%)	161 (37.3%)	49 (11.3%)	58 (13.4%)	19 (4.4%)	2.17	1.16

*(1) Strongly Disagree, (2) Disagree, (3) Neutral, (4) Agree, and (5) Strongly Agree.

As stated elsewhere, while students are selected based on a threshold level and order of merit after the benchmark for student entry requirements, teachers are not selected based on their proficiency in English. This is confirmed in Item (31), where 55.1% of the respondents thought their English was better than that of their teachers. Only 26.7% admitted that their content teachers are more proficient in English. This finding again goes with the germane mainstream literature in Tunisia or other socio-cultural contexts. In Kuteeva et al. (2015), students reported high proficiency in English. Emirati respondents in Solloway (2016) reported how their non-Anglophone teachers struggled with English. Students found this a demotivating factor that affects teaching effectiveness and students’ attendance and academic performance. In Tunisia, teachers lacked adequate mastery of English and the pedagogical training to teach in EMI programs. In addition, given the recent adoption of EMI and the long years of French-medium instruction, it is difficult to find content teachers with high proficiency in English. Hence, unless the content teachers are already proficient or taking the initiative to improve their English, they cannot keep up with the students’ seemingly good English. Surprisingly, and unlike cases reported in the literature from other non-Anglophone contexts, students did not find dealing with textbooks in English difficult (item 32), nor did they have problems structuring their thoughts in English (33). Both questions have a low mean of 2.77 and 2.17 and a relatively low SD of 1.01 and 1.16, respectively. Students in the sample do not have significant linguistic challenges in using EMI since 70.9% of respondents disagreed with the statement. This goes contrary to the evidence of recent literature (Rakhshandehroo, 2017; Ali, 2020; Lei and Hu, 2022; Oraif and Alrashed, 2022; Volchenkova and Al-Darraji, 2022) where students reported problems in using English in the classroom context. Again, this can be explained by the threshold level of English to enter EMI programs in the public sector.

### 4.4. EMI classroom practices

**Figure 1:**
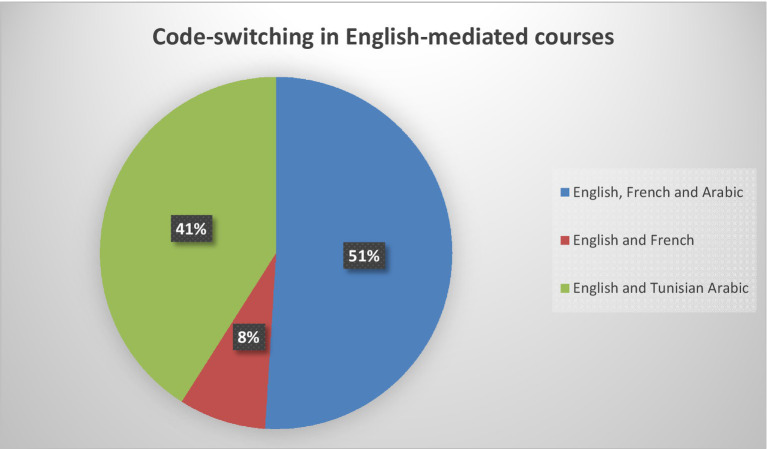
What languages do teachers mix? (Item 35, Number of respondents, 324).

This section answers the fourth research question about translanguaging practices during English-mediated courses. More specifically, it reports on the teaching practices and classroom dynamics with a focus on classroom talk and interactions that take place. Overall, the students in this study perceived English as the language of academia and the language of their careers in the future. Most of them anticipated the need for English in any career path they would endeavor. Answering Item (34), ‘Only English is used during Instructional hours’, a majority of 74.7% of students reported translanguaging and language shuttle in the EMI classroom setting. In a related issue, Item (35), ‘what languages do teachers mix?’, the students’ responses were quite surprising to me as I did not expect the use of Tunisian Arabic in a formal teaching context. Figure 1 reports on the parallel language use in the EMI classroom. Approximately 51% of respondents stated that teachers alternate English, French, and Arabic, 41% thought teachers mix English and Tunisian Arabic, and only 8% stated that content teachers used English and French. Already, code-switching is a common practice in the Tunisian classroom setting. Bach Baoueb and Toumi (2012) stated that code-switching is a common practice both in the classroom and outside the classroom. Teachers too shuttled between English, French, and Tunisian Arabic. It is evident that the teacher’s ultimate objective is to achieve the communicative goals of the lesson and engage students. The code of communication seems to be secondary, although English is the code of English-mediated courses (Table 5).

**Table 5:** Current practices of EMI.

Questionnaire Item	Yes	No	Maybe
34. Only English is used during Instructional hours.	110 (25.3%)	324 (74.7%)	00 (0%)
36. English language learning objectives are included in the EMI curriculum	181 (41.7%)	52 (12%)	201 (46.3%)
37. Student ask teachers to translate into French or Arabic if they do not understand the lecture in English.	266 (61.3%)	94 (22.4%)	71 (16.4%)
38. Language competencies are evaluated in EMI programs.	190 (44%)	113 (26%)	131 (30%)

Similarly, when responding to Item (38), ‘Students ask teachers to translate into French or Arabic if they do not understand the lecture in English’, most respondents (61.3%) agreed with the statement. They reported asking their teachers if they need to grasp academic knowledge in English. Again, a classroom observation validated this. While attending a marketing EMI course titled Operational Marketing and Branding, I noticed this translanguaging in action. Although the bulk of the lesson was in English and the reference textbooks were also in English, the content teacher and the students were code-switching and using English, French, and Tunisian Arabic, often in one sentence. This was confirmed by the classroom observation where the teachers and the students code-switched flexibly. Extract 1 shows how the classroom setting is used as a translanguaging space. The excerpt below is taken from a marketing course where the content teacher opted for English as a medium of instruction. Yet, as shown in the extract, the classroom exchange was multilingual.

Extract 1:

Teacher: What is the difference between brand features and brand benefits?

[students are silent].

Student A: Features are *les avatanges du marque* (French for advantages of the brand) and.

Student B: Characteristics of the brand *mɑʕnɛ:ha* (Tunisian Arabic for ‘it means/they are’) (Student C overlaps)… *les valeurs du marque* [French for ‘brand value’].

Teacher: Features give you technical information about the product. Benefits tell you how [pause] your life will be better with the brand or product. Ummmm, for example, a brand watch is made of gold that is a feature. It makes look rich; that is a benefit.

[Students whisper to each other in Tunisian Arabic].

Teacher: Ok, what are features and benefits in Arabic?

[Students are silent].

Teacher: Features *hu:mɑ* [Tunisian Arabic for ‘they are’] *mumeizet aw mi:zet*. Benefits *yaʕni mana:fiʕ* [Standard Arabic for ‘that means benefits’].

Students: laughter.

Student C: [laughing] *Monsieur en Francais,* please. [Sir, in French, please].

Teacher: [laughing as he responds in French] *Ils sont les caracteristiques et les advantages du marque* [French for: They are the characteristics and the advantages of the brand].

The aforementioned extract showcases the integrated and parallel language used in the teacher–student negotiation of meaning. In this extract, the teacher introduces ‘brand features and brand benefits’ as two key concepts in their course to students who are majoring in Marketing. The extract is a typical example of linguistic bricolage where translanguaging, as a practice, shapes the EMI classroom experience. In this context, students drew from their combined linguistic and communicative repertoires in English, French, and Arabic (Tunisian uncodified dialect) to negotiate academic content. Students A and B, in the aforementioned extract, resorted to French and, to a lesser extent, Tunisian Arabic, when their lexical repertoire in English failed them. This resonates with Lewis et al.’s (2012) and Reynolds’ (2022) accounts of the strategic use translation within translanguaging educational settings. In these EMI contexts, academic content in the weaker academic language (i.e English) is often translated into the student’s stronger language (e.g. French or Arabic) to ensure understanding of content. Student C’s request to explain in French is perhaps indicative that French is the student’s stronger academic language. By shuttling between languages without triglossic functional separation, the content teacher and the students cooperated to create a space for trilingual interaction. In addition, it is evident that the teacher and students, flexibly and freely, activated the whole array of linguistic repertoire. It is also evident that students code-switched at will switching off a code before switching on another. This can only help in maximizing the understanding of academic content and the engagement of students which can be otherwise curbed by the limited linguistic repertoire in a monolingual context and rigid monolingual classroom settings. This flexibility of the teacher when they go on to translate upon the request of the student, serves pedagogical and motivational ends. Here, translation, as plan B, plays a pedagogic role in accomplishing the task of introducing new concepts and keeping the students engaged in the academic content. It falls into the category of translation labeled “translation of subject-related terminology, which can be identified as a scaffolding approach to help pupils complete tasks undertaken in the classroom” (Lewis et al., 2012, p. 296). Such an encounter is a typical example of flexible trilingualism and permeability of language use where the code takes a backseat to the accomplishment of the pedagogical goals of the academic course. The extract also gives confirmatory evidence to Bash Baoueb and Toumi (2012), who stated that code-switching in Tunisian high education contexts, especially in economics and management classes, is tolerated “when confined to communicative goals” (p. 279). This argument still holds true now, and I believe, it is more prevalent now with the introduction of EMI programs. It also shows the students’ struggle with English. This seems to put a double burden on the students. They need to take care of English while struggling with academic content. Badwan (2019) nicely articulated this when she states that Tunisian students still “grapple with language at a time when they are expected to learn advanced specialized knowledge” (p. 30).

On other occasions, there were many instances of negative transfer from French and cases of cross-linguistic influence and borrowing with Englishization. The teacher often intervened to correct the student’s language use mistakes. This is illustrated by the heteroglossic exchange reported in Extract 2.

Extract 2:

Teacher: How brands are made? Is every product a brand?

Student A: The product can be tranformated (sig) to a brand with the stages of branding.

Teacher: [correcting the student] transformed not transformated. Ok, now, remind me of the stages of brand development.

[Students’ talk is overlapping while they recall the stages].

In this extract, three points need to be highlighted. First, how the student borrowed and derived from the French noun ‘transformation’ (transform) and then ‘Englishized’ using the particle ‘ed’ in a clear case of negative language transfer and wrong word derivation. Second, we see the teacher not interfering to correct the student until they finish their idea. Third, playing the role of a language teacher, the content teacher offers to correct the student’s use of English.

## 5. Conclusion

English-medium instruction is a leading trend in research in higher education and it gained more momentum. This study offered a Tunisian perspective on the flowing literature across the globe. It investigated students’ perception of EMI in Tunisian HEIs Institutions as well as the challenges they encounter. It also investigated translanguaging practices within the EMI classroom setting. Overall, the students had a fairly positive attitude toward the implementation of EMI and its emergence in Tunisian universities as well as using it in their future careers. Although the students reported great confidence in their English, they qualified their content teachers’ English as below par. Classroom practices showed a great level of translanguaging and code-switching. This was explained by the triglossic situation in the country.

In order to counter the pedagogical and educational inadequacies which arise from the challenges reported in this study, the following pedagogical implications and education policy requirements are perhaps in place. Since teachers’ proficiency in English was a reiterated challenge for students, multilingual and pedagogic teacher training of content teacher is a burning issue. Since teachers are accustomed to teaching in French and they transpose their teaching know-how from French to English, training in EMI pedagogy can perhaps sensitize them to the pedagogical differences. as a threshold level in English is mandatory for students to enter these elite schools, a similar threshold level to teach EMI classes should be imposed on teachers. Authorities should take the teacher’s mastery of English into account when recruiting teachers for tenured positions at these elite schools In-service professional training for content lecturers can boost the effectiveness of EMI course delivery. For example, content lecturers should be encouraged financially to enroll in English certification programs. Finally, given the urgency to swap to EMI, authorities should accelerate the transition to English in other HEIs, and it should not be left to teacher initiative and willingness.

This study explored the status of English and its use as a medium of instruction in tertiary higher education in Tunisia. Although this study tried to report on the EMI status in Tunisia, it does not claim to be inclusive of all facets of EMI programs in the country. Although the study reports interesting findings, it also has limitations in terms of research design. Although the online questionnaire and especially snowball sampling allow access to a big mass of the studied population, the study is prone to the limitations of this research method. The questionnaire model, especially the structured version, does not leave room for the respondents to clarify their options or elaborate on them. This makes the data lack qualitative in-depth. This limitation should be minded when making generalizations, especially the unexpected high level of proficiency the respondents had reported. In addition, the elite nature of the schools to which the respondents are affiliated tends to slightly limit the representativeness of the population.

In order to have a comprehensive understanding of EMI in Tunisia, further research can tackle the following research areas. It would be worthwhile to take the perspective of content teachers. Hence, a qualitative ethnographically oriented study is perhaps an interesting research avenue. Issues such as teachers’ perception of EMI, the pedagogical strategies, institutional support provisions, pedagogical differences between EMI and French medium instruction, EMI eligibility requirements, challenges, and coping strategies are confirmed research niches. In addition, studying the transition from French-medium instruction to EMI would yield interesting results. Finally, a needs analysis study that targets the pedagogical needs of content teachers would help us know how best to support EMI content teachers.

## Data availability statement

The original contributions presented in the study are included in the article/supplementary material, further inquiries can be directed to the corresponding author.

## Author contributions

The author confirms being the sole contributor of this work and has approved it for publication.

## Conflict of interest

The author declares that the research was conducted without any commercial or financial relationships that could be construed as a potential conflict of interest.

## Publisher’s note

All claims expressed in this article are solely those of the authors and do not necessarily represent those of their affiliated organizations, or those of the publisher, the editors and the reviewers. Any product that may be evaluated in this article, or claim that may be made by its manufacturer, is not guaranteed or endorsed by the publisher.

## Footnotes

^1^https://www.ef.com/wwen/epi/ (accessed 29 September 2022).

^2^Official statistics from the Ministry of Higher Education in Tunisia.
